# An Immunochromatographic Test Strip and Its Application in Rapid Screening of Pepper Mild Mottle Virus

**DOI:** 10.3390/bios16030135

**Published:** 2026-02-25

**Authors:** Xin Yang, Kelei Han, Wenyao Zhang, Chen Zhang, Rui Fan, Tingtao Chen, Yan Jin, Jiashuo An, Zichen Zhu, Xiaolong Shao, Guoliang Qian, Dankan Yan, Limin Wang

**Affiliations:** 1 State Key Laboratory of Agricultural and Forestry Biosecurity, College of Plant Protection, Nanjing Agricultural University, Nanjing 210095, China; 2023102033@stu.njau.edu.cn (X.Y.); zhangwenyao1231@163.com (W.Z.); 19855132090@163.com (C.Z.); fanandrui@163.com (R.F.); 2023802227@stu.njau.edu.cn (T.C.); 2025102038@stu.njau.edu.cn (Y.J.); a13687658123@163.com (J.A.); zhuzichen@stu.njau.edu.cn (Z.Z.); 2021067@njau.edu.cn (X.S.); glqian@njau.edu.cn (G.Q.); 2 Institute of Plant Protection and Agro-Products Safety, Anhui Academy of Agricultural Sciences, Hefei 230031, China; hankeleihz@163.com

**Keywords:** pepper mild mottle virus, coat protein, monoclonal antibodies, immunochromatographic strip

## Abstract

The quality and productivity of peppers have been severely impacted by the pepper mild mottle virus (PMMoV). To effectively control the transmission and damage caused by PMMoV, it is crucial to establish a simple, rapid, and field-applicable detection method. In this study, the coat protein of PMMoV was induced expression as an immunogen. After cell fusion and subcloning, a total of 10 hybridoma cell lines that stably secrete the PMMoV monoclonal antibody were screened. Through antibody pairing and screening, using monoclonal antibody 4D7 as the capture antibody and 3B9 as the detection antibody, an immunochromatographic test strip for PMMoV was established. Under ideal conditions, the test strip’s color development indicated that its detection limit for the target protein was 1 ng/mL, and the result was obtained in 10 min. The findings of field testing and specific detection demonstrated that this test strip could reliably identify PMMoV and it is capable of distinguishing between different disease samples collected in this study. It is anticipated that this test strip will be able to offer services for PMMoV field detection.

## 1. Introduction

Peppers (*Capsicum annuum* L.) are economically important Solanaceae vegetable crops worldwide, as they contain a wide range of biologically active compounds with broad therapeutic potential against human diseases [1,2]. However, this crop is highly susceptible to various pathogenic infections, including fungi, bacteria, and viruses [3,4]. Among these pathogens, viral agents are the most devastating to pepper [5,6].

Pepper mild mottle virus (PMMoV) is a rod-shaped virus with a positive-sense RNA genome of about 6.3 kb, belonging to the genus *Tobamovirus.* It primarily infects solanaceous plants and is one of the major viruses on peppers [7,8]. PMMoV causes mild to severe symptoms on capsicum, including leaf mosaic, mottling, puckering, vein thickening, stunting, and upward cupping of the leaves [9]. Additionally, PMMoV causes modest symptoms that may remain undetected in the field and only become evident during the fruiting stage [10]. PMMoV can be transmitted not only by seed dispersal and sap rubbing, but also can enter the human body with diseased peppers, and after being digested and excreted, it still has infestation activity and possesses a great potential for spreading [11,12]. PMMoV has been reported to pose a global threat to pepper production, with incidence reaching as high as 95%, causing 75% to 95% yield losses [13]. In order to effectively control the spread and damage of this disease, the first thing to do is to monitor the disease accurately and in a timely manner, so the development of a simple, sensitive, and rapid field detection technology is very necessary.

At present, domestic and foreign detection techniques for PMMoV include electron microscopy, molecular biology, serological detection techniques, etc. For example, Filipic used transmission electron microscopy (TEM) to observe the presence of PMMoV virus particles and their morphological changes after cold atmospheric plasma (CAP) treatment [14]. Additionally, RT-PCR, RT-RAA-LFS, and ELISA are currently widely used (Table 1). Priyankaben detected PMMoV in pepper seeds using RT-PCR and, considering its mode of transmission, suggested the use of an established procedure to detect PMMoV from the seeds [15]. Cao utilized RT-RAA in combination with lateral flow chromatography strips (LFS) to simultaneously detect RNA-containing PMMoV and DNA-containing anthrax genomes in the field [16]. Wang used DAS-ELISA and RT-PCR to test fresh infected tissues of peppers and compared the two methods [12]. However, these techniques require specialized personnel, high equipment costs, complex sample handling, and demanding environmental conditions [17].

Colloidal gold immunochromatography strip (CGIS) is a rapid, low-cost, sensitive, and accurate solid phase labeling technique that obviates the need for specialized instrumentation and skilled operators [18,19,20], allowing for the on-the-spot monitoring of hazardous substances across diverse fields [21]. Currently, CGIS technology has been widely applied to the detection of plant virus diseases such as areca palm velarivirus 1, soybean mosaic virus, rice stripe virus, banana bract mosaic virus, and green mottle mosaic virus, etc. [22,23,24,25,26]. Table 1 presents the comparison of various methods for PMMoV detection, highlighting that existing detection methods are dependent on instruments and with time-consuming operations. In contrast, CGIS does not require professional instruments, and it has the advantages of simple operation. Immunoassays based upon the specific recognition of antigens by antibodies are widely used owing to their rapidity, ease of use, and affordability [21,27,28]. DAS-ELISA based on the PMMoV monoclonal antibody has been published, but this method usually requires the antibodies to undergo treatments such as conjugation with alkaline phosphatase. As far as we know, there is still no published reports on the preparation of CGIS using PMMoV monoclonal antibodies to date.

In this study, the coat-protein-encoding region of PMMoV was cloned into expression vector system and expressed in BL21 (DE3), a protease-deficient strain of *Escherichia coli*. Then, we employed the PMMoV coat protein expressed by *E. coli* as an immunogen in mice for the production of monoclonal antibodies. Using hybridoma cell lines via cell fusion, a total of 10 monoclonal antibodies were prepared, and it was found that antibodies 4D7 and 3B9 form a good matching antibody pair for sandwich immunoassays. After optimization, the test strip was able to accurately detect PMMoV, and it can distinguish the distinct disease samples collected in this study. This test strip has a high sensitivity for detecting PMMoV, and its detection limit for the target protein reached 1 ng/mL in 10 min. This detection method enables rapid and large-scale sample testing on site and has a promising application prospect.

## 2. Materials and Methods

### 2.1. Materials

#### 2.1.1. Materials Related to PROTEIN EXPRESSION

The *Escherichia coli* BL21 (DE3) strain used in this experiment was stored by the plant quarantine and bacteriology laboratory of Nanjing Agricultural University. Taq enzyme was purchased from Kangwei Century Biotechnology Co., Ltd. (Taizhou, China). Isopropyl-β-D-1-thiogalactopyranoside (IPTG) (purity ≥ 99%) was purchased from Solarbio Science & Technology Co., Ltd. (Beijing, China). 4 × Protein SDS PAGE Loading Buffer was purchased from TaKaRa Biomedical Technology Co., Ltd. (Beijing, China). Protein Electrophoresis Precast Gel and MOPS were purchased from ACE Biotechnology Co., Ltd. (Nanjing, China).

#### 2.1.2. Materials Related to Antibody Screening

The SP2/0 cell line was stored and cultured by the plant quarantine and bacteriology laboratory of Nanjing Agricultural University. The animal experiments were conducted at the Hubei BioNT Experimental Center. Freund’s Complete Adjuvant (FCA), Freund’s Incomplete Adjuvant (FIA), 30% hydrogen peroxide (386790-M), tetramethylbenzidine (TMB) (purity ≥ 99%), liquid paraffin, dimethyl sulfoxide (DMSO) (purity ≥ 99.9%), and glycol (PEG1500) were purchased from Sigma Chemical Co. (St. Louis, MO, USA). Hypoxanthine aminopterin thymidine (HAT), hypoxanthine thymidine (HT), and Dulbecco’s Modified Eagle Medium (DMEM) were provided by Gibco (Grand Island, NY, USA). Horseradish peroxidase-labeled goat anti-mouse IgG conjugate (HRP-GaMIgG) was acquired from Zhuyan Biological Technology Co., Ltd. (Nanjing, China). Fetal bovine serum (FBS) was provided by Lanzhou Minhai Biological Engineering Co., Ltd. (Lanzhou, China). The Antibody Subtype Identification Kit was purchased from Sino Biological Inc. (Beijing, China).

#### 2.1.3. Materials Related to the Preparation of Test Strips

Tetrachloroauric acid solution (purity ≥ 99.995%) and trisodium citrate (purity ≥ 98%) were purchased from Sigma Chemical Co. (St. Louis, MO, USA). The PVC base plate (MT101B), binding pad (8964), sample pad (GF-08), absorbent pad (H-3), test strip cassettes, and nitrocellulose (NC) membranes used in the preparation of the test strips were purchased from Shanghai Jiening Biotech Co. (Shanghai, China).

#### 2.1.4. Source of Virus

Tobacco mosaic virus (TMV), cucumber green mottle mosaic virus (CGMMV), pepper mild mottle virus (PMMoV), tomato brown rugose fruit virus (ToBRFV), and tobacco mild green mosaic virus (TMGMV) were preserved by the Anhui Academy of Agricultural Sciences and stored at −80 °C for later use.

#### 2.1.5. Primers Involved in the Experiment

The experiment involved specific detection, with the specific primers used listed in (Table S1). Initial denaturation for 30 s at 98 °C, 10 s denaturation at 98 °C, 5 s of annealing at 60 °C, and 10 s extension at 72 °C followed by a final extension at 72 °C for 1 min were performed. Thirty-five cycles of denaturation and extension were performed. Amplicons were analyzed on a 1% agarose gel.

### 2.2. Construction of pET28a-CP System

Extraction of total RNA from PMMoV-infected plants and reverse transcription to cDNA were performed. Primer pairs with built-in XhoI and BamHI restriction enzyme cutting site were designed based on the sequence of the PMMoV *cp* gene in Genbank, (F: gtggtggtggtggtgctcgagAGGAGTTGTAGCCCAGGTGAGTC; R: cagcaaatgggtcgcggatccATGGCTTACACAGTTTCCAGTGC), and using this primer, the PMMoV *cp* gene was amplified using the above cDNA as a template. The prokaryotic expression vector pET28a was double digested with XhoI and BamHI, respectively, and then ligated to the recovered fragment of the PMMoV *cp* gene with ligase, and subsequently transferred into *E. coli* strain DH5α. Positive colonies were selected to extract plasmids for transfer into *E. coli* strain BL21.

### 2.3. Induced Expression of PMMoV Recombinant Protein

Firstly, a single bacterial colony was cultured in 3 mL of Luria–Bertani (LB) broth with 100 ppm kanamycin at 37 °C for 5–6 h. And then 200 μL of bacterial solution was taken to a new 20 mL of Luria–Bertani (LB) broth with 100 ppm kanamycin at 37 °C and 200 r/min shaking. The optical density at 600 nm (OD600) of the bacterial culture reached 0.4 to 0.6, isopropyl-β-D-1-thiogalactopyranoside (IPTG) was added to the culture medium (the final concentration was 1 mM), and at 16 °C, 200 r/min induction was cultured overnight. After induction, we collected the bacteria and discarded the supernatant, added 1 mL of 1 × PBS for sonication, centrifuged at 6000 rpm for 10 min, and recorded the supernatant as “NPE”. To the precipitate, 100 μL of 1 × PBS (containing 8 M urea) was added and resuspended and mixed, labeled as “DPE”. 9 μL of “NPE” and “DPE” were added to 3 μL of 4 × Protein SDS-PAGE Loading Buffer and boiled for 10 min. Finally, it was analyzed by 12.5% SDS-polyacrylamide gel electrophoresis (SDS-PAGE).

Further bulk expression was performed by the same method as for small volume expression, expanded to a 300 mL system. After collection of bacteria, 20 mL of 1 × PBS was added for sonication. The precipitate was resuspended with 1 × PBS (containing 8 M urea), collected for gradient dialysis of target proteins into the appropriate buffer, and purified. The soluble protein was labeled with His-tag using a nickel column, and the eluted products were analyzed by SDS-PAGE and ultrafiltered for storage and use.

### 2.4. Preparation of the Monoclonal Antibody Against PMMoV

PMMoV coat protein was used as immunogens, and Freund’s complete adjuvant was used for the first immunization and Freund’s incomplete adjuvant for subsequent immunizations. The immunogen was mixed with the adjuvant in equal volume, after fully emulsifying the mixture. Five BALB/C female mice, aged 6–8 weeks old, were immunized, and each mouse was injected with 200 µL. A total of five immunizations were performed, with three weeks between the second and first immunizations and two weeks between the remaining immunizations. After the third immunization, one week after each immunization, venous blood from the tail of the mice was taken to detect the immunization effect by ELISA assay, and one mouse with the best immunization effect was selected for the next cell fusion experiment.

The SP2/0 cells were resuscitated one week prior to performing cell fusion and cultured with DMEM solution containing 20% fetal bovine serum. Three days before cell fusion, the selected mice were intensively immunized (200 μL of immunogen without adjuvant). Feeder cells were prepared from unimmunized 6–8-week-old BALB/C female mice the day before fusion. For cell fusion, mouse spleens were taken and ground with DMEM, and splenocytes were obtained by centrifugation to remove the supernatant and mixed well with SP2/0 cells, and the fusion of the two types of cells was induced with PEG1500, after which the reaction was terminated with DMEM. Fused cells with complete culture medium were suspended and evenly spread into 96-well cell plates. The cells were screened by HAT and HT screening and subcloned by multiple limited dilution methods, and hybridoma cells with high titer were obtained. High-purity and specific mAb were obtained from the hybridoma supernatant.

### 2.5. Preparation of Colloidal Gold Immunochromatography Strip

#### 2.5.1. Preparation of Colloidal Gold

Colloidal gold particles (40 nm in diameter) were prepared using the trisodium citrate reduction method, as described by Contreras-Trigo et al. [29]. Earlier studies in the laboratory have conducted transmission electron microscopy (TEM) validation on colloidal gold [30,31]. We optimized the protocol on the basis of previous studies; referring to the validation by Rivera et al. [32], the maximum absorption peak of 40 nm colloidal gold is approximately at 528 nm, and the absorption peak detection method was subsequently adopted for the validation of colloidal gold in all subsequent experiments. The detailed experimental procedures for the preparation of colloidal gold are as follows:

We added 100 mL of triple-distilled water to a clean conical flask, then added the rotor, and placed the conical flask on the magnetic stirring heater. We performed heating until there were small bubbles around the rotor to start the rotor and until there were bubbles rising from the bottom of the bottle and water mist at the mouth of the bottle. We quickly added 1 mL of 1% concentration of chloroauric acid solution and 1 mL of 1% concentration of trisodium citrate solution. The solution was timed from black to red for 5 min, cooled at room temperature, dispensed into wide-mouth glass bottles, and stored at 4 °C in a refrigerator protected from light. The finally prepared colloidal gold showed a wine-red color with a maximum absorption peak at approximately 528 nm (Figure S1).

#### 2.5.2. Preparation of Colloidal Gold-Labeled Monoclonal Antibodies

Firstly, the pH of 1 mL of colloidal gold solution was adjusted to 8.5 using 0.1 M K_2_CO_3_. Secondly, an appropriate amount of monoclonal antibody dissolved in 0.01 M PBS buffer was added, and the nanoparticles were shaken and mixed for twenty minutes so that the nanoparticles were in full contact with the antibody. Next, the filtered 10% BSA solution was added, with 100 μL of BSA per 1 mL of colloidal gold solution, and shaken for two minutes. After one hour of standing at room temperature, the mixture was centrifuged at 4 °C for 20 min (10,000 rpm). After careful removal of the supernatant, the precipitate was resuspended in 0.5% boric acid buffer, and the resuspension could be stored at 4 °C or spread on a binding pad for subsequent experiments.

#### 2.5.3. Assembly of Test Strips

The test strip consists of five parts: base plate, sample pad, colloidal gold pad (binding pad), nitrocellulose membrane (NC membrane), and absorbent pad. Two types of lines were delineated on the NC membrane; line C is the quality control line, delineated with broad-spectrum antibodies; line T is the detection line, delineated with specific antibodies. The paper strips were assembled in order, and the PVC base plate was placed at the bottom. According to the direction of lateral flow from front to back, the sample pads, binding pads, nitrocellulose membranes, and absorbent pads were glued to the PVC base plate. After the test strip assembly was completed, the CM4000 strip cutter purchased from BioDot, Inc. (Irvine, CA, USA) was used to cut the assembled test strip into 3.5 mm width and mount the card case for dry and sealed storage.

### 2.6. Optimization of Working Conditions for Test Strips

Different working conditions of the test strips have a great influence on the final detection results. Including the pH of the colloidal gold solution [33] when it is too high or too low can cause the antibody to denature. Antibody concentration is also a very important influencing factor [34], with excessive amounts causing false positive. Considering that the final result is determined by the deposition of colloidal gold particles on the nitrocellulose membrane, the type of the nitrocellulose membrane is also an important optimization direction.

## 3. Results

### 3.1. Induced Expression of PMMoV Recombinant Protein

**Figure 1:**
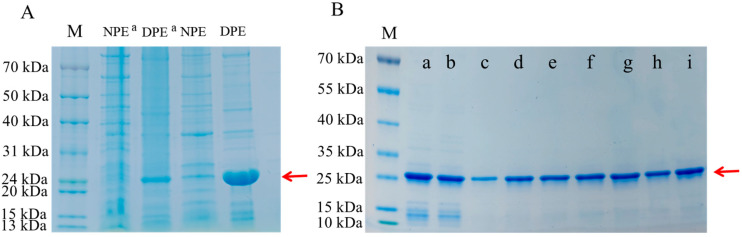
Induced expression of PMMoV recombinant protein. (**A**) Small-scale expression of PMMoV recombinant protein. ^a^, without IPTG induction. “NPE” represents the protein in the supernatant, “DPE” represents the protein in the precipitate. (**B**) Large-scale expression and purification of PMMoV recombinant protein. a–i, representing the protein eluted from the sample each time with 500 microliters of elution solution during protein purification. The red arrow pointed to the target band.

In order to prepare immunogens, the pET28a-PMMoV CP plasmid was transformed into BL21 (DE3) receptor cells to induce expression of small amounts of proteins. The proteins were analyzed by SDS-PAGE and stained with Coomassie brilliant blue. The results showed that the target antigenic proteins were expressed in both the supernatant protein “NPE” and the inclusion body protein “DPE”, but mainly in the inclusion body protein (the size of the target band was approximately 26 kDa) (Figure 1A). A large number of proteins were further induced to be expressed. The inclusion body proteins were combined with nickel column purification after gradient dialysis, and the eluted products were analyzed by SDS-PAGE. The results showed that the target bands were clear and there were no obvious heterogeneous bands (Figure 1B). The protein concentration was measured after ultrafiltration to prepare for use.

### 3.2. Pairing of the Monoclonal Antibodies

After cell fusion and subcloning, a total of 10 hybridoma cell lines that stably secrete the PMMoV monoclonal antibody were screened, and through the determination of antibody subtypes, mAbs 1F12, 5B12, 4E6, and 6B8 were IgG1; mAbs 4D7, 3E1, 4D10, and 5C7 were IgG2a; and mAbs 3B9 and 2B2 were IgG2b. Subsequently, the coat protein was immobilized on ELISA plates, and the binding ability of these 10 antibodies to it was detected via indirect ELISA, with the results presented in (Figure S2).

**Figure 2:**
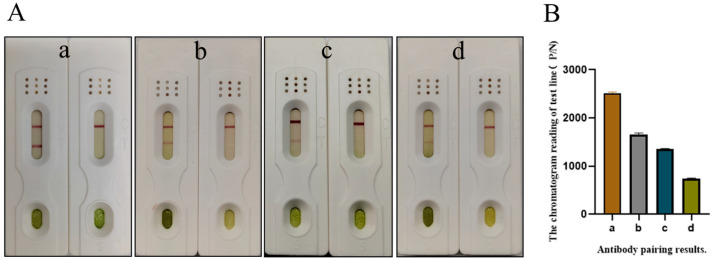
The results of antibody pairing. (**A**) The results of test strips; for each group, the left side corresponds to the detection of positive samples, and the right side to that of negative samples. (**B**) The results of P/N ratios. a, 4D7 as the capture antibody and 3B9 as the detection antibody; b, 3E1 as the capture antibody and 3B9 as the detection antibody; c, 1F12 as the capture antibody and 3B9 as the detection antibody; d, 5B12 as the capture antibody and 3B9 as the detection antibody.

After antibody pairing experiments; the positive and negative signal values of the paired test strips were read using a portable chromatographic reader, and the P/N ratios were subsequently calculated. Ultimately, the use of 3B9 as the detection antibody and 4D7, 1F12, 3E1, and 5B12 as individual capture antibodies yielded favorable P/N ratios with negligible false positive signals. The pairing results for test strip are shown in (Figure 2A), and the P/N ratios calculated via the chromatographic reader are shown in the same (Figure 2B). In addition, we weighed the same amount of PMMoV, TMV, TMGMV, CGMMV, and ToBRFV, as well as healthy leaf samples. Subsequently, we ground each sample separately, diluted them by a factor of 1000, and then immobilized the diluted samples onto ELISA plates. Indirect ELISA was used to assess the specificity of the five selected antibodies, and the results are shown in (Figure S3). The experiments of test strip reading analysis and specificity detection were conducted with three replicates. In summary, 4D7 and 3B9 exhibited favorable pairing efficacy and specificity; thus, these two antibodies were ultimately selected for the preparation and optimization of test strips.

### 3.3. Optimization of the Immunochromatographic Test Strip

#### 3.3.1. Optimization of the Nitrocellulose Membrane

In this experiment, five different nitrocellulose membranes (CN140, JN140, Pall120, Pall170, FF120) were used to detect the color development of the test strips. The deepest T-lines were observed when testing diseased samples with the Pall120 or CN140, and no false positives were detected in healthy leaves (Table S2). After testing the stability of the test strips, Pall120 was finally selected as the nitrocellulose membrane for this test strip.

#### 3.3.2. Optimization of the Test Line

The concentration of the T-line antibody on the nitrocellulose membrane has a significant impact on the final color reaction result of the test strip, with excessive amounts wasting antibodies and resulting in false positives. In order to determine the optimal capture antibody concentration for the T-line, we set up five concentration gradients (2 mg/mL, 2.5 mg/mL, 3 mg/mL, 3.5 mg/mL, and 4 mg/mL) to test the same sample. The results showed that the color reaction was the best when the T-line concentration was 3 mg/mL and without false positive phenomenon (Table S2).

#### 3.3.3. Optimization of Surfactants

As a non-ionic surfactant, Tween-20 can reduce the flow resistance of the buffer solution in the test strip, can increase the chromatographic speed and uniformity, and can make the test results more accurate and reliable. In this experiment, 0.1%, 0.2%, 0.3%, 0.4%, and 0.5% of Tween-20 were added to the buffer solution, respectively. During the test strip chromatography process, the chromatography speeds and final chromatographic results of the test strips under different conditions were compared. The results showed that as the amount of Tween-20 added increased, the color of the T- line became darker and the chromatographic speed continuously accelerated. When the dosage of Tween-20 was 0.3%, 0.4%, and 0.5%, the test strip chromatography was fast, and the color of the T-line showed no significant change. Therefore, we finally decided to add 0.3% of Tween-20 to the buffer solution (Table S2).

### 3.4. Analytical Evaluation of the Immunochromatographic Test Strip

#### 3.4.1. Sensitivity of the Immunochromatographic Test Strip

**Figure 3:**
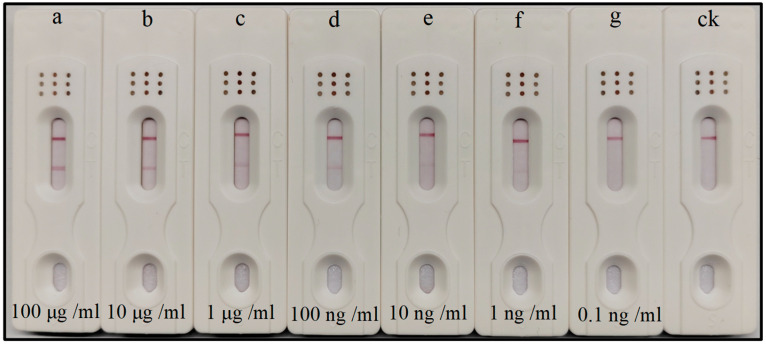
Sensitivity analyse of the test strip. a–g, representing the protein concentration as 100 μg/mL, 10 μg/mL, 1 μg/mL, 100 ng/mL, 10 ng/mL, 1 ng/mL, 0.1 ng/mL; ck, buffer solution.

A series of PMMoV recombinant proteins with concentration gradients were tested by PMMoV test strips, and color intensity was measured 10 min after samples were added. The results showed that a bright red band was detected for the positive result, with no detectable band for the negative result at PMMoV recombinant protein concentrations as low as 1 ng/mL (Figure 3).

#### 3.4.2. Specificity of the Immunochromatographic Test Strip

**Figure 4:**
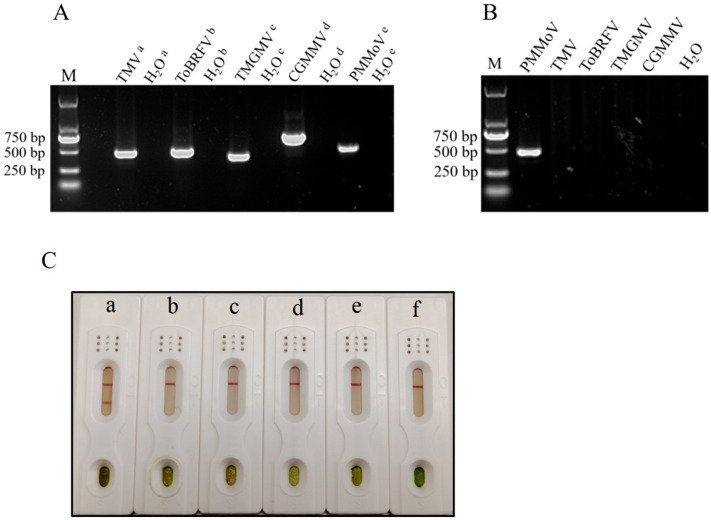
Specificity analysis of the test strip. (**A**) Different disease samples were verified by PCR using their respective specific primers. ^a^, specific primers of TMV, the size of the band was 444 bp. ^b^, specific primers of ToBRFV, the size of the band was 460 bp. ^c^, specific primers of TMGMV, the size of the band was 370 bp. ^d^, specific primers of CGMMV, the size of the band was 654 bp. ^e^, specific primers of PMMoV, the size of the band was 450 bp. (**B**) Detected plant samples using PMMoV-specific primers. (**C**) The specific detection results of the test strip. a–f, representing PMMoV, TMV, ToBRFV, TMGMV, CGMMV and a healthy pepper leaf (control), respectively.

Specificity is a crucial assessment aspect when detecting PMMoV. Here, we selected four *Tobamoviruses* commonly found in pepper plants: TMV, CGMMV, ToBRFV, and TMGMV [26,35,36,37]. Infected leaf samples from these viruses were used to test the specificity of PMMoV immunochromatographic test strip. Simultaneously, the RT-PCR method was used to confirm the presence of the virus in each plant sample. The samples were tested using their respective specific primers, with water serving as the negative control. The results showed that viruses were present in the plant samples (Figure 4A). Further testing of plant samples was conducted using PMMoV-specific primers; the results showed that there was no PMMoV in these plant samples (Figure 4B). The test strip results showed that the C line and T line of the tested samples from PMMoV-infected plant developed a color response, whereas samples from leaves infected with TMV, ToBRFV, TMGMV, and CGMMV and healthy leaf showed no color at the T line (Figure 4C). The above experiments demonstrated that the PMMoV test strip is capable of distinguishing between different disease samples collected in this study.

#### 3.4.3. Stability of the Immunochromatographic Test Strip

**Figure 5:**
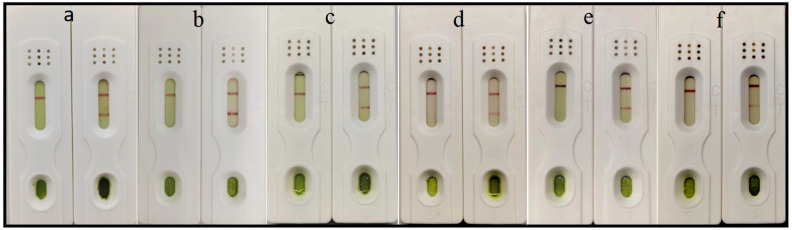
Stability assessment of the immunochromatographic test strip. On the left side of each group was the test result for healthy leaf, and on the right side was the test result for diseased sample. a–f, 30 days; 90 days; 150 days; 210 days; 270 days; 330 days.

Each batch of test strips was separately placed in an aluminum foil bag containing desiccant and then stored in a dry environment. We regularly took out the test strips for testing; the test strips produced highly stable and valid results for about one year (Figure 5).

#### 3.4.4. Evaluation of Leaf Effects on Test Strips

The complex matrix present in plant leaves can cause interference in the detection by the test strips [38]. In order to detect the effect of substances in the leaves on the results of the test strips, we placed 0.05 g, 0.1 g, 0.2 g, and 0.3 g of healthy and diseased leaves of peppers in a grinding bag, after which 2 mL buffer solution was added to each grinding for test strip assays. The results indicated that as the number of diseased leaves increased, the T line on the test strip became more intensely colored. However, when the quantity of leaves exceeded 0.2 g, the chromatography speed slowed down, and the leaves significantly affected the background of the test strips. Therefore, selecting 0.1 g of leaves for testing was more appropriate (Table 2).

### 3.5. Sample Testing

#### 3.5.1. Inoculated Samples Testing

**Figure 6:**
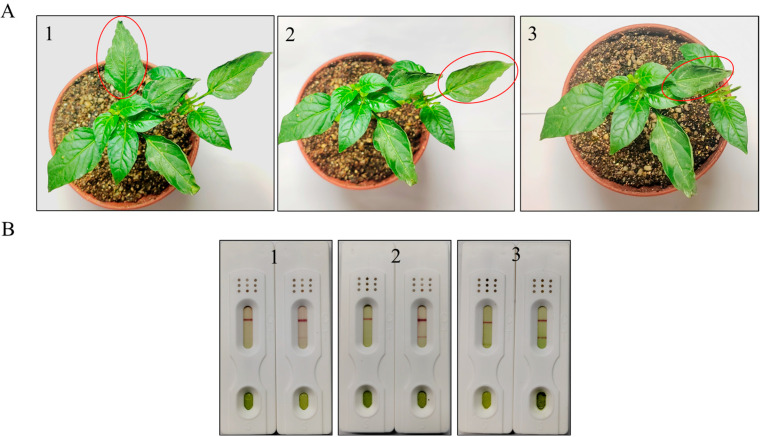
Inoculated samples testing. (**A**) Samples of plant inoculation. The red circle indicated the test sample. (**B**) The results of the test strip. (1) Test result of leaf test strip after inoculation for 2 days; (2) Test result of leaf test strip after inoculation for 4 days; (3) Test result of leaf test strip after inoculation for 6 days.

In this experiment, the PMMoV-infected samples were ground and then inoculated through mechanical friction. At 2 days, 4 days, and 6 days after inoculating, test strips were used to separately test the inoculated samples and the healthy samples. The circled leaves were the test subjects after inoculation (Figure 6A). On the left side of each group, the test strip represents the test results for the healthy samples, while the test strip on the right side represents the test results for the inoculated samples. The test results are shown in (Figure 6B); four days after the inoculation, the test strip showed a clearly positive band.

#### 3.5.2. Field Sample Testing

**Figure 7:**
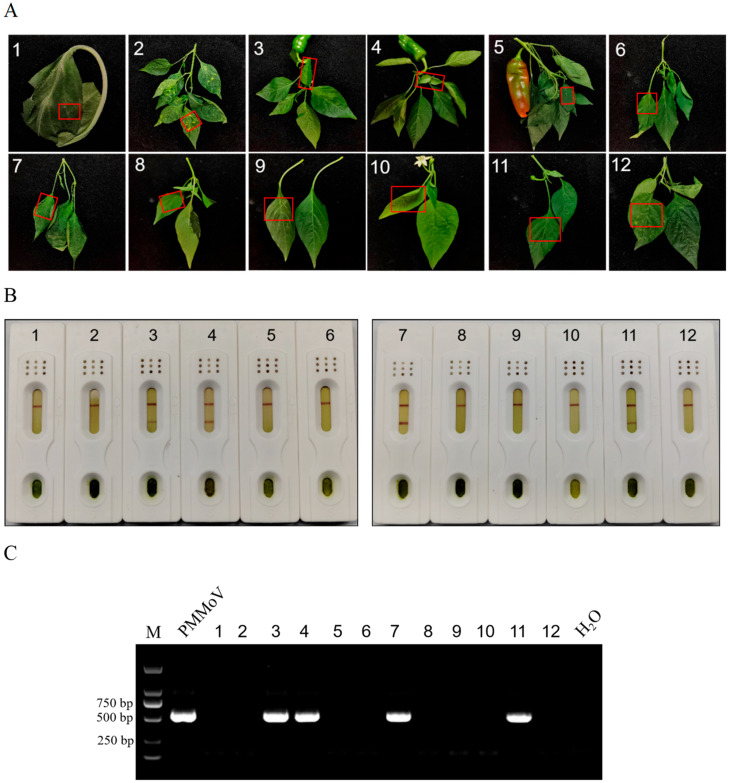
Field sample testing. (**A**) Pepper leaves in the field. The red box indicated the sampling area. (**B**) Test results obtained using the test strip detection method for the samples. (**C**) Test results for samples using the RT-PCR detection method. 1–12 representing different plant samples.

The accuracy of the test strips was tested by using the field samples of peppers from He County, Ma’anshan City, Anhui Province. We took 2 × 2 cm pieces of pepper leaves and placed them into the grinding bag, added buffer solution, and ground thoroughly. Then, we conducted the test strip detection. We concurrently performed RT-PCR testing on the same plant sample and compared the results with those from the test strip. Field samples are shown in (Figure 7A), using the test strip method; samples 3, 4, 7, and 11 tested positive, while all other samples tested negative (Figure 7B). Detection was performed using the RT-PCR method, with PMMoV disease samples serving as positive controls and water as negative controls; the results were consistent with those obtained from the test strip (Figure 7C). In summary, this test strip exhibited high accuracy.

## 4. Conclusions

An immunochromatographic test strip detecting PMMoV was developed, and under ideal conditions, its detection limit for the target protein reached 1 ng/mL. The developed detection assay has favourable specificity and stability; moreover, the entire process took only 10 min from sample collection to the visible presentation of results. In addition, this detection method does not require instruments; it is expected to achieve the goal of rapid field detection of PMMoV and provide services for real-time monitoring of PMMoV. Meanwhile, considering that our detection target is a plant virus, and given that during the field testing process the rate of virus release in sample pre-processing is closely related to the detection speed of the test strip, we will subsequently focus more on improving virus release efficiency by optimizing the working system of the test strip. In addition to this, in subsequent research, we may need to obtain coat proteins from the other four tobamoviruses (TMV, ToBRFV, TMGMV, CGMMV) to quantify the specificity of the test strip.

## Figures and Tables

**Table 1:** Comparison of different methods for detecting PMMoV.

Method	Detection Time	Is Sample Processing Required ?	Sensitivity	Instrumentation	Is This Technique Laboratory Environment-Dependent?	Reference
RT-RAA-LFS ^a^	25 min	RNA extraction and cDNA synthesis	0.32 copies/μL of target genes	portable toolbox: includes a metal bath, vortex oscillator, etc.	no	[16]
RT-PCR	more than 1 h	RNA extraction and cDNA synthesis	0.008 µg of fresh infected tissue	vortex oscillator, the instrument about Polymerase Chain Reaction, etc.	yes	[12]
DAS-ELISA	more than 6 h	-	39 µg of fresh infected tissue	washing instrument, constant-temperature incubator, etc.	yes	[12]
CGIS	10 min ^b^	-	1 ng/mL of target protein	observation with the naked eye	no	this study

^a^ One-tube reverse transcription-recombinase aided amplification (RT-RAA) combined with lateral flow strip (LFS) assay. ^b^ The entire process took only 10 min from sample collection to the visible presentation of results.

**Table 2:** Leaf effects on test strips.

Factor		The Coloration Degree of the T Line	Chromatographic Speed	Background of Separation Technique
Healthy Leaves	0.05 g	−	fast	/
	0.1 g	−	fast	/
	0.2 g	−	slow	*
	0.3 g	−	slow	**
Diseased leaves	0.05 g	++	fast	/
	0.1 g	+++	fast	/
	0.2 g	+++	slow	*
	0.3 g	+++	slow	**

“−” and “+” represent negative and positive results, respectively. “+++” is darker than “++”. “/” and “*” indicate no effect on chromatographic background and a green chromatographic background, respectively. “**” is darker than “*”.

## Data Availability

The data presented in this study are available on request from the corresponding authors. The data are not publicly available due to ethical constraints.

## Supplementary Materials

The following supporting information can be downloaded at: https://www.mdpi.com/article/10.3390/bios16030135/s1, Table S1: Primers involved in the experiment, Table S2: Effect of different parameters on the test strip results, Figure S1: Color characterization and absorption peak determination of colloidal gold, Figure S2. Determination of titer of mAbs by indirect ELISA, Figure S3. Specificity detection of different antibodies.
