# Supplementary material for: An Immunochromatographic Test Strip and Its Application in Rapid Screening of Pepper Mild Mottle Virus
Source: Biosensors (Basel). 2026 Feb 25;16(3):135. doi: 10.3390/bios16030135 (PMC13024521; doi:10.3390/bios16030135)
Supplement: Supplementary file 1 [file biosensors-16-00135-s001.zip › biosensors-4119868-supplementary.pdf]

An Immunochromatographic Test Strip and its application in rapid screening of Pepper mild mottle virus

Xin Yang[1,+], Kelei Han[2,+], Wenyao Zhang[1], Chen Zhang[1], Rui Fan[1], Tingtao Chen[1], Yan Jin[1], Jiashuo An[1], Zichen Zhu[1], Xiaolong Shao[1], Guoliang Qian[1], Dankan Yan[2,*], Limin Wang[1,*]

1 State Key Laboratory of Agricultural and Forestry Biosecurity, College of Plant Protection, Nanjing Agricultural University, Nanjing 210095, China; 2023102033@stu.njau.edu.cn (X.Y.); zhangwenyao1231@163.com (W.Z.); 19855132090@163.com (C.Z.); fanandrui@163.com (R.F.); 2023802227@stu.njau.edu.cn (T.C.) ; 2025102038@stu.njau.edu.cn (Y.J.); a13687658123@163.com (J.A.); zhuzichen@stu.njau.edu.cn (Z.Z.); 2021067@njau.edu.cn (X.S.); glqian@njau.edu.cn (G.Q.)

2 Institute of Plant Protection and Agro-Products Safety, Anhui Academy of Agricultural Sciences, Hefei, 230031, China; hankeleihz@163.com (K.H.)

* Correspondence: dkyan2011@163.com (D.Y.); wlm@njau.edu.cn (L.W.)

+ Xin Yang and Kelei Han are authors contributed equally to this work

Supporting

Table S1. Primers involved in the experiment

Virus [a]	Primer [b]	Sequence 5’-3’ [c]	Amplicon size (bp)
TMV	TMV-F	GTTCTTGTCATCAGCGTGGG	444
	TMV-R	CAAGTTGCAGGACCAGAGGT	
ToBRFV	ToBRFV-F	ATGTCTTACACAATCGCAACTC	460
	ToBRFV-R	CCATTGTAAACCGGATGCAC	
TMGMV	TMGMV-F	CAAATGCATTGGGTAACCAG	370
	TMGMV-R	TGTGGTCCAGACAAGTCCAC	
CGMMV	CGMMV-F	CGTGGTAAGCGGCATTCTAAACCTC	654
	CGMMV-R	CCGCAAACCAATGAGCAAACCG	
PMMoV	PMMoV CP-F	CCAGTGCCAATCAATTAGTG	450
	PMMoV CP-R	AGTTGTAGCCCAGGTGAGTC	

[a] TMV=Tobacco mosaic virus, ToBRFV=Tomato brown rugose fruit virus, TMGMV=Tobacco mild green mosaic virus, CGMMV=cucumber green mottle mosaic virus, PMMoV=Pepper mild mottle virus.

[b] F=forward primer, R=reverse primer.

[c] Primers were designed independently based on Genebank accession numbers.

Table S2. Effect of different parameters on the test strip results.

Optimization facter		healthy leaves [a]	diseased leaves [a]
Nitrocellulose membrane	CN140	-	+++
	JN140	-	+
	Pall120	-	+++
	Pall170	-	++
	FF120	-	++
T-line concentration	2 mg/ml	-	+
	2.5 mg/ml	-	+
	3 mg/ml	-	+++
	3.5 mg/ml	+	+++
	4 mg/ml	+	++++
Tween-20 (%)	0.1	-	++
	0.2	-	++
	0.3	-	+++
	0.4	-	+++
	0.5	-	+++

"+" represents the color depth of the test strip detection line; "++++" indicates the darkest color of the detected line; "+++" is darker than "++"; "++" is darker than "+"; "- "represents no color.

[a], the size of the leaf was approximately 2×2 cm.

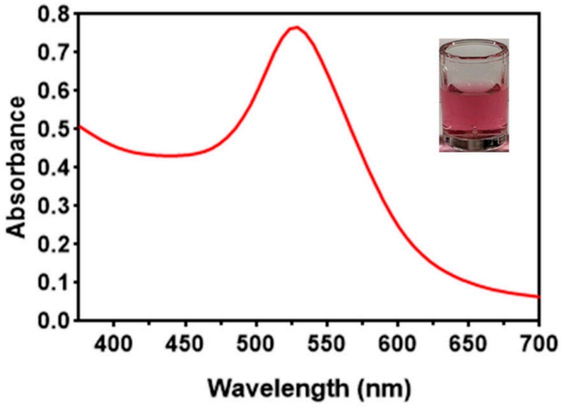

Figure S1. Color characterization and absorption peak determination of colloidal gold..It showed a wine-red color with a maximum absorption peak at approximately 528 nm.

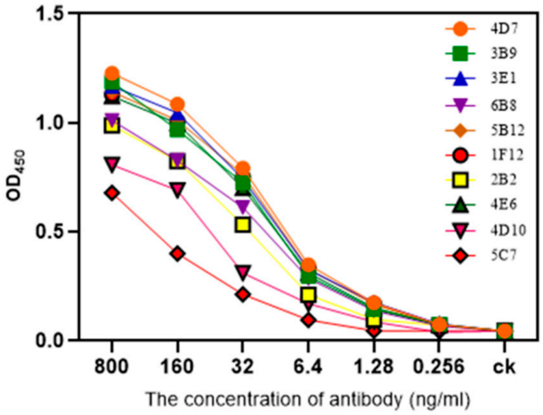

Figure S2. Determination of titer of mAbs by indirect ELISA.(the coat protein immobilized on ELISA plates)

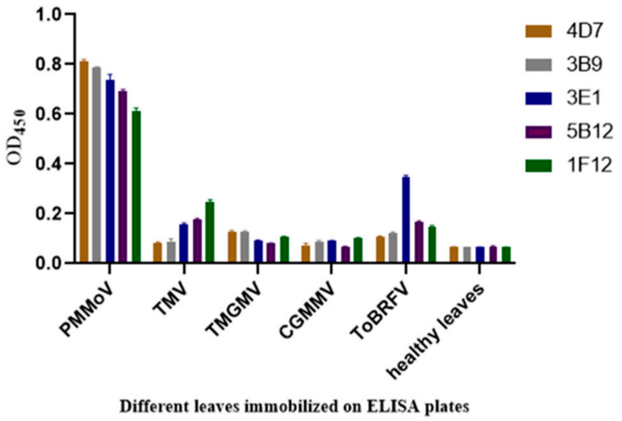

Figure S3. Specificity detection of different antibodies.
